# Correction: Badaraev et al. Surface Modification of Electrospun Bioresorbable and Biostable Scaffolds by Pulsed DC Magnetron Sputtering of Titanium for Gingival Tissue Regeneration. *Polymers* 2022, *14*, 4922

**DOI:** 10.3390/polym16162372

**Published:** 2024-08-22

**Authors:** Arsalan D. Badaraev, Dmitrii V. Sidelev, Anna I. Kozelskaya, Evgeny N. Bolbasov, Tuan-Hoang Tran, Alexey V. Nashchekin, Aleksandra S. Kostina, Anna B. Malashicheva, Sven Rutkowski, Sergei I. Tverdokhlebov

**Affiliations:** 1 Weinberg Research Center, School of Nuclear Science & Engineering, National Research Tomsk Polytechnic University, 30, Lenin Avenue, 634050 Tomsk, Russia; 2 Ioffe Institute, 26, Polytekhnicheskaya Street, 194021 St. Petersburg, Russia; 3 Institute of Cytology RAS, 4, Tikhoretsky Avenue, 194064 St. Petersburg, Russia; aleksandrakostina1991@gmail.com (A.S.K.);

## Addition of an Author

Aleksandra S. Kostina was not included as an author in the original publication [1]. The corrected Author Contributions statement appears below. The authors state that the scientific conclusions are unaffected. This correction was approved by the Academic Editor. The original publication has also been updated.

**Author Contributions:** A.D.B.: Investigation, Writing—Original Draft, Visualization; D.V.S.: Investigation; A.I.K.: Writing—Review and Editing; E.N.B.: Resources; T.-H.T.: Investigation; A.V.N.: Investigation; A.B.M.: Investigation, Supervision; A.S.K.: Investigation, S.R.: Writing—Review and Editing, Supervision, Validation; S.I.T.: Conceptualization, Supervision, Project Administration, Funding Acquisition. All authors have read and agreed to the published version of the manuscript.
