# Supplementary material for: Surface Modification of Electrospun Bioresorbable and Biostable Scaffolds by Pulsed DC Magnetron Sputtering of Titanium for Gingival Tissue Regeneration
Source: Polymers (Basel). 2022 Nov 15;14(22):4922. doi: 10.3390/polym14224922 (PMC9698656; doi:10.3390/polym14224922)
Supplement: Supplementary file 1 [file polymers-14-04922-s001.zip › polymers-2021741-supplementary.pdf]

Support Information of: Surface modification of electrospun bioresorbable and biostable scaffolds by pulsed DC magnetron sputtering of titanium for gingival tissue regeneration

Arsalan D. Badaraev[1], Dmitrii V. Sidelev[1], Anna I. Kozelskaya[1], Evgeny N. Bolbasov[1], Tuan-Hoang Tran[1], Alexey V. Nashchekin[3], Anna B. Malashicheva[2], Sven Rutkowski[1*], Sergei I. Tverdokhlebov[1*]

[1] National Research Tomsk Polytechnic University, 30, Lenin Avenue, 634050, Tomsk, Russian Federation
[2] Institute of Cytology RAS, 4, Tikhoretsky Avenue, 194064, St. Petersburg, Russian Federation
[3] Ioffe Institute, 26, Polytekhnicheskaya Street, 194021, St. Petersburg, Russian Federation
* Correspondence: rutkowski_s@tpu.ru (S.R.), tverd@tpu.ru (S.I.T.)

Supporting Figures:

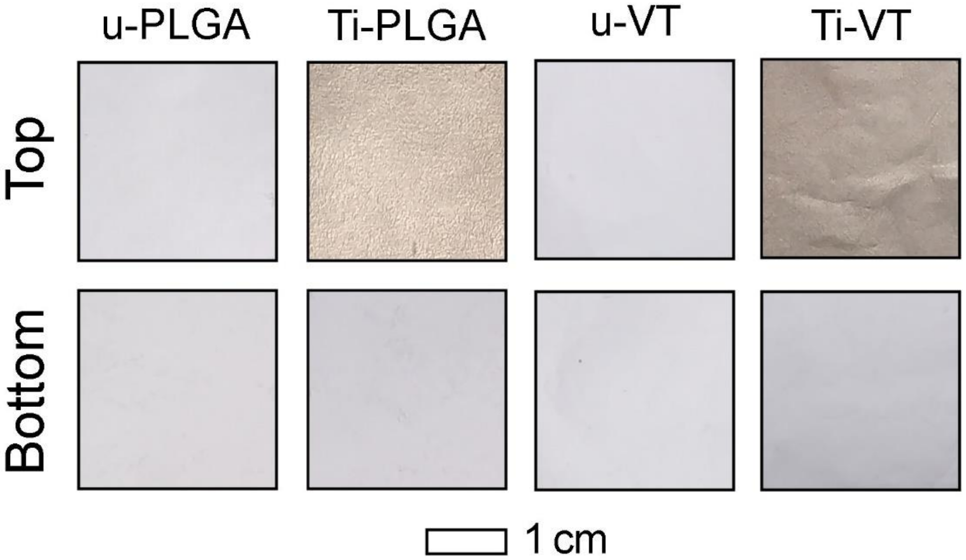

Figure S1. Photographs of the top and bottom surfaces of the unmodified (u-PLGA and u-VDF-TeFE) and the surface-modified (Ti-PLGA and Ti-VDF-TeFE) scaffolds made from poly(lactide-co-glycolide) (PLGA) and vinylidene fluoride – tetrafluoroethylene copolymer (VDF-TeFE).

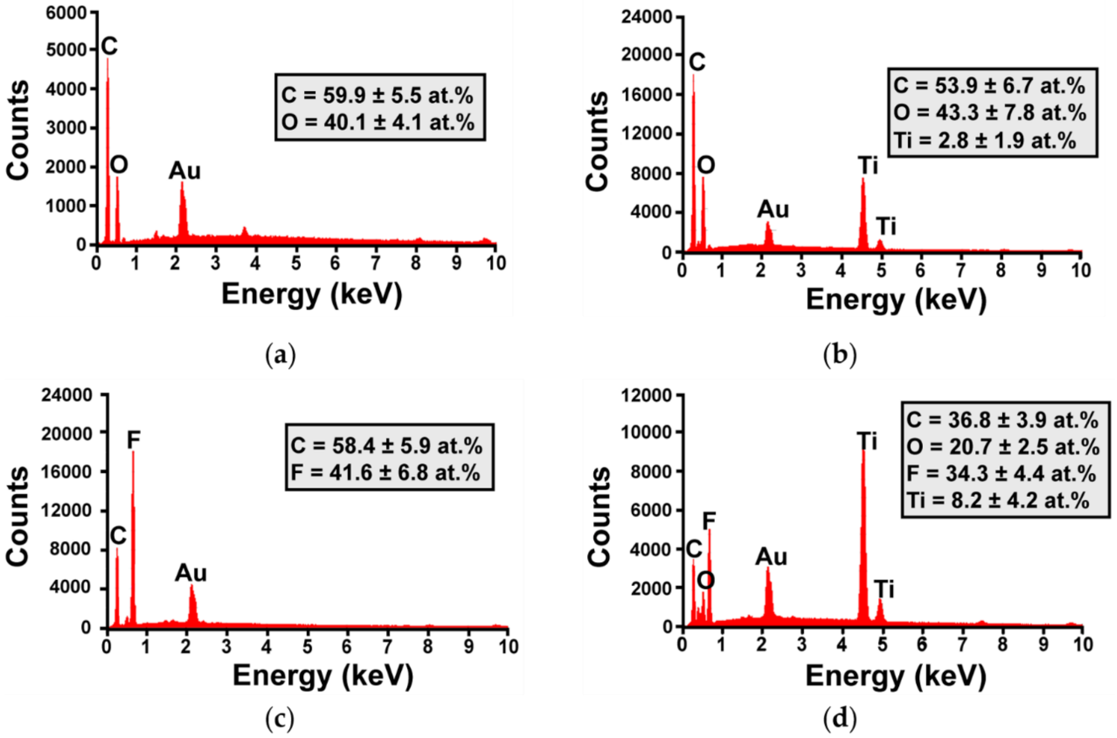

Figure S2. EDX spectra of the unmodified (u-PLGA and u-VDF-TeFE) and the surface-modified (Ti-PLGA and Ti-VDF-TeFE) scaffolds made from poly(lactide-co-glycolide) (PLGA) and vinylidene fluoride – tetrafluoroethylene copolymer (VDF-TeFE): (**a**) u-PLGA, (**b**) Ti-PLGA, (**c**) u-VDF-TeFE, (**d**) Ti-VDF-TeFE. All elemental concentration values are presented in atomic percent (at.%).

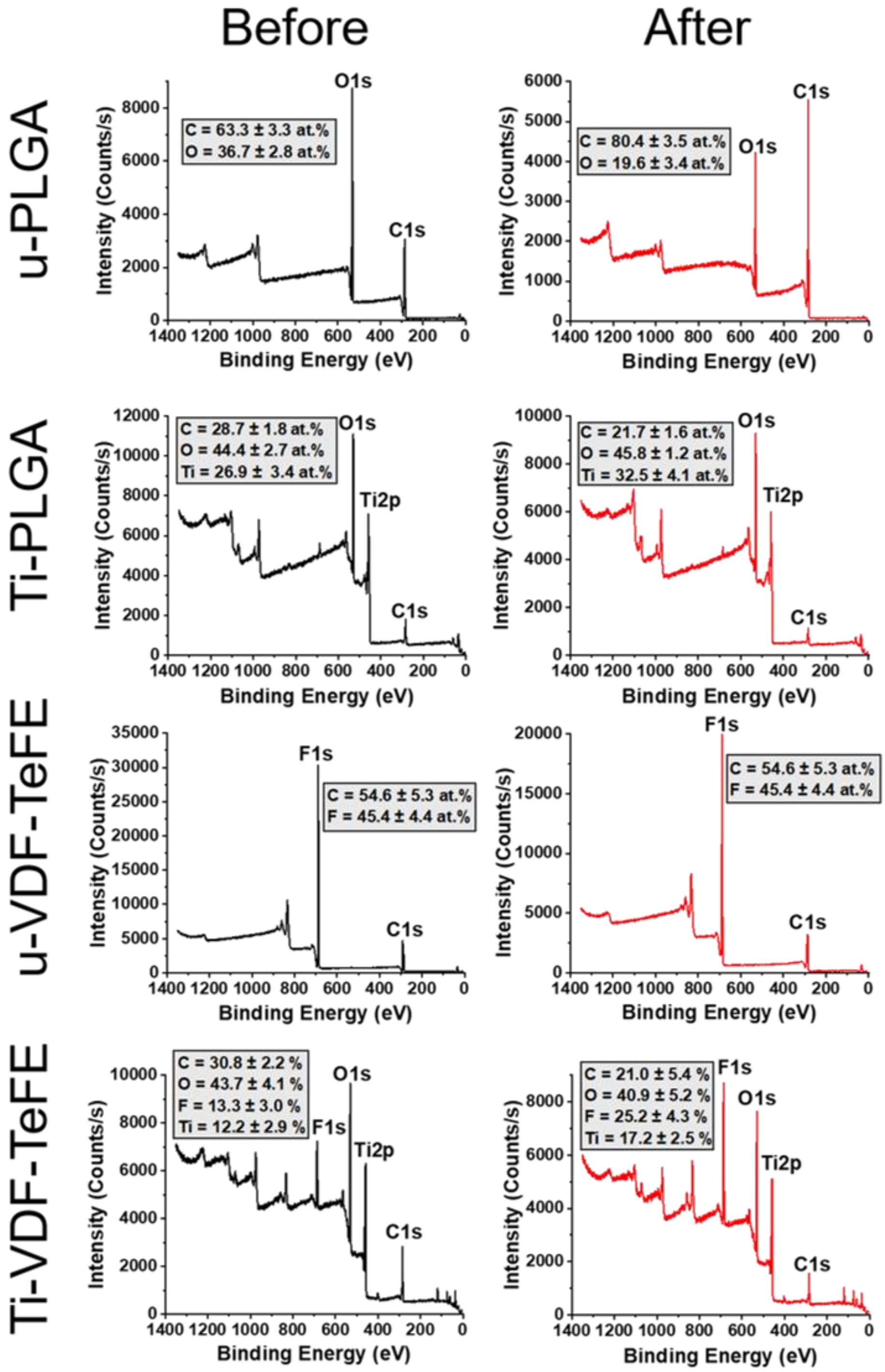

Figure S3. XPS survey spectra before ion beam treatment (scaffold sample surface cleaning procedure by argon plasma) on the left side and XPS survey spectra after ion beam treatment on the right side of the unmodified (u-PLGA and u- VDF-TeFE) and the surface-modified (Ti-PLGA and Ti-VDF-TeFE) scaffolds made from poly(lactide-co-glycolide) (PLGA) and vinylidene fluoride – tetrafluoroethylene copolymer (VDF-TeFE). All elemental concentration values are presented in atomic percent.

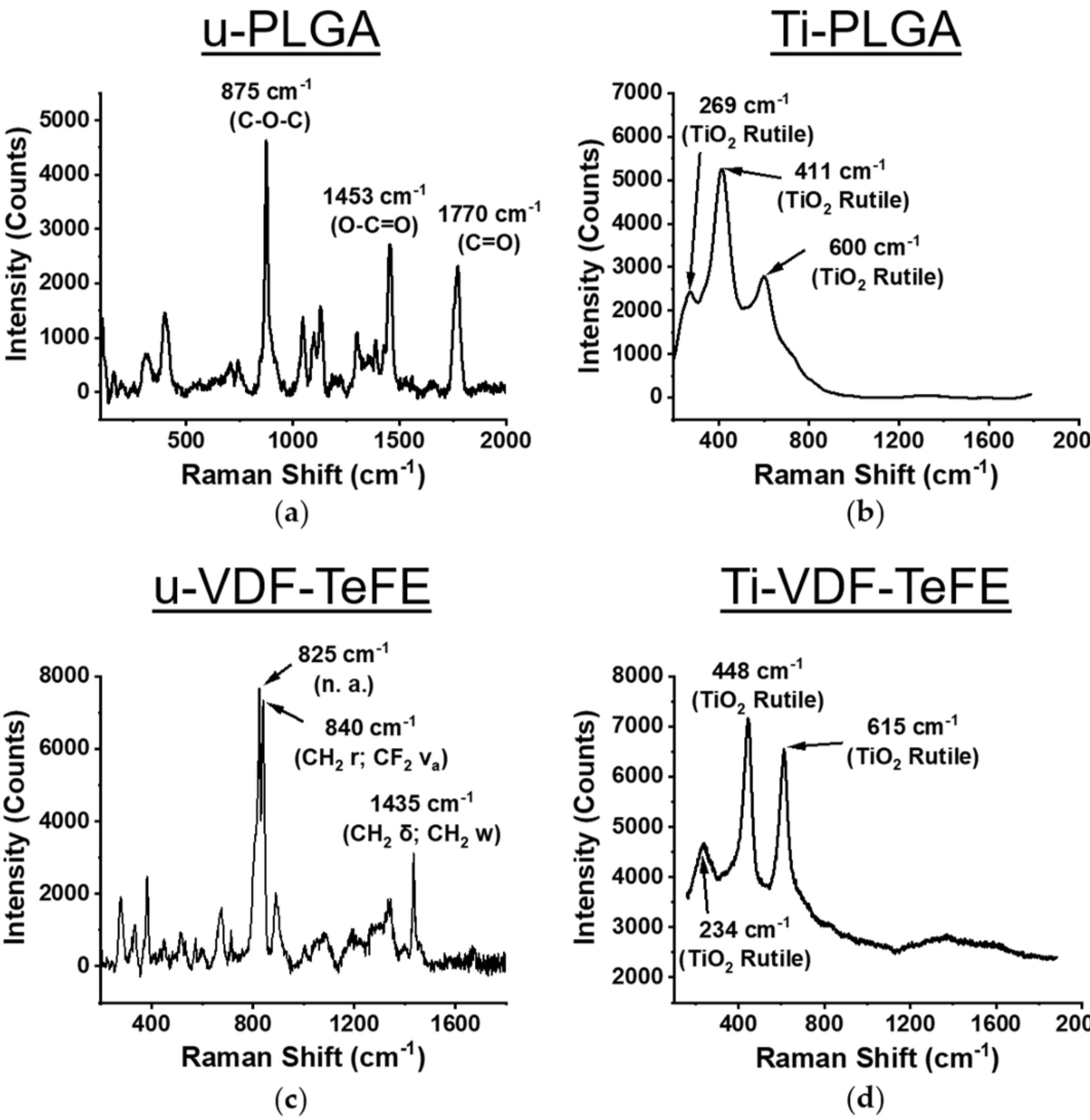

Figure S4. Raman spectra of the unmodified (u-PLGA and u-VDF-TeFE) and the surface-modified (Ti-PLGA and Ti-VDF-TeFE) scaffolds of poly(lactide-co-glycolide) (PLGA) and vinylidene fluoride – tetrafluoroethylene copolymer (VDF-TeFE): (**a**) u-PLGA, (**b**) Ti-PLGA, (**c**) u-VDF-TeFE, (**d**) Ti-VDF-TeFE. Abbreviations in the spectra of (**a**) and (**c**) are as follows: v_a - antisymmetric stretching, δ - scissoring, w - wagging, r - rocking, n.a. - not assigned.

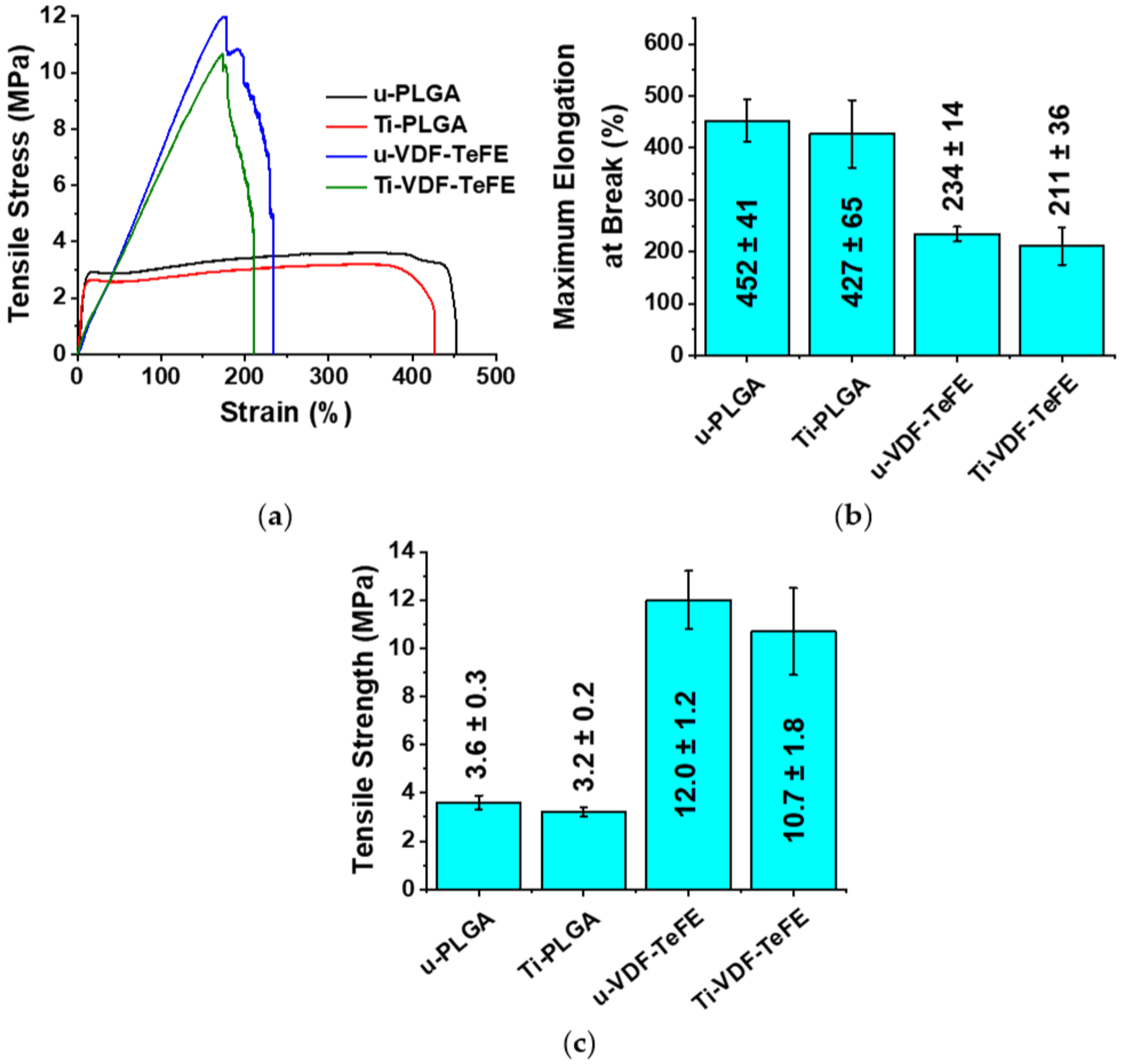

Figure S5. Mechanical properties of the unmodified (u-PLGA and u-VDF-TeFE) and the surface-modified (Ti-PLGA and Ti-VDF-TeFE) scaffolds made from poly(lactide-co-glycolide) (PLGA) and vinylidene fluoride – tetrafluoroethylene copolymer (VDF-TeFE): (**a**) stress-strain diagrams, (**b**) maximum tensile strength and (**c**) maximum elongation at break.

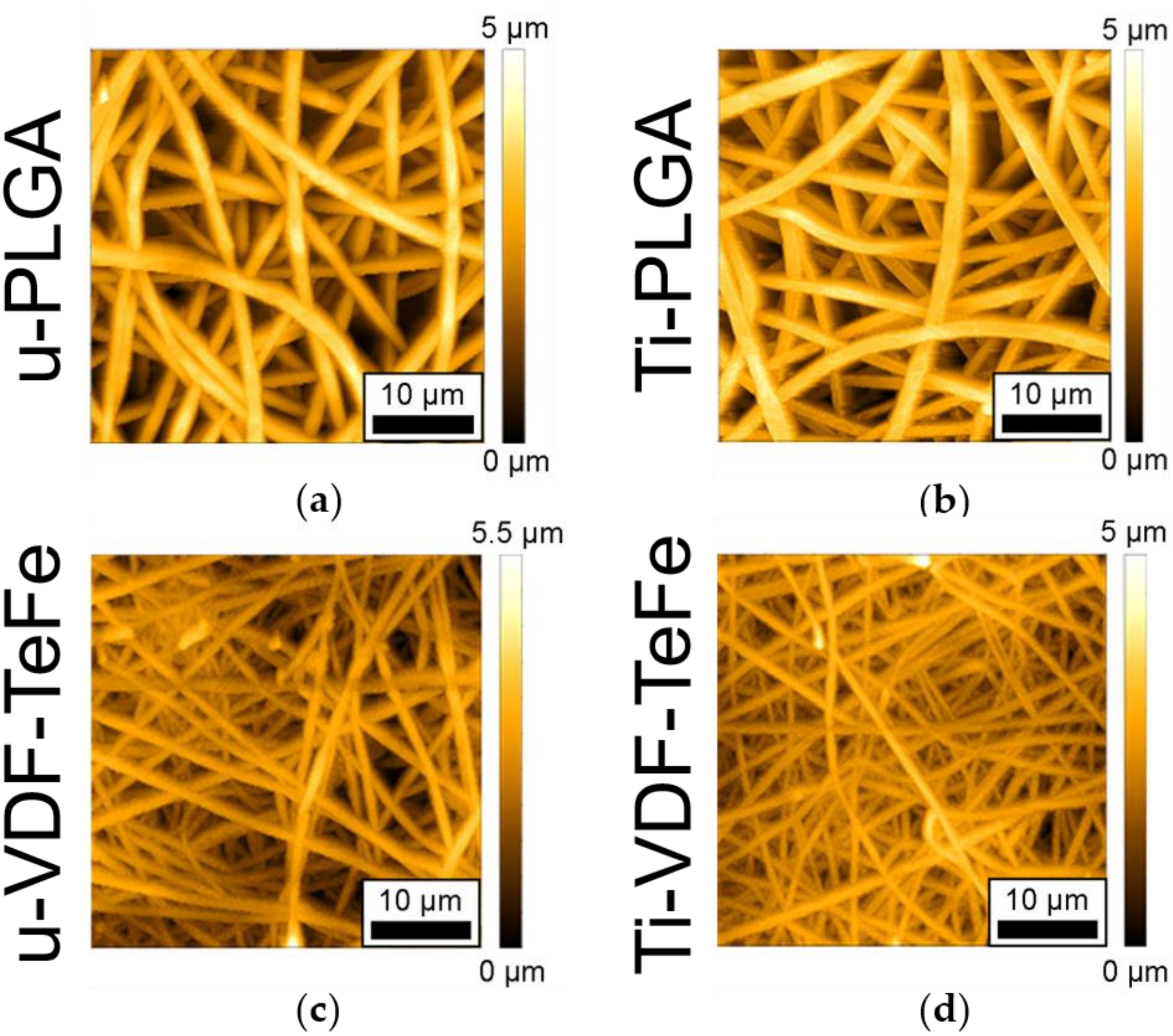

Figure S6. Atomic force microscopy (AFM) micrographs obtained on a 40×40 µm^2 scanning area of the unmodified (u-PLGA, u-VDF-TeFE) and the surface-modified (Ti-PLGA, Ti-VDF-TeFE) scaffolds made from poly(lactide-co-glycolide) (PLGA) and vinylidene fluoride – tetrafluoroethylene copolymer (VDF-TeFE): (**a**) u-PLGA, (**b**) Ti-PLGA, (**c**) u-VDF-TeFE, (**d**) Ti-VDF-TeFE.

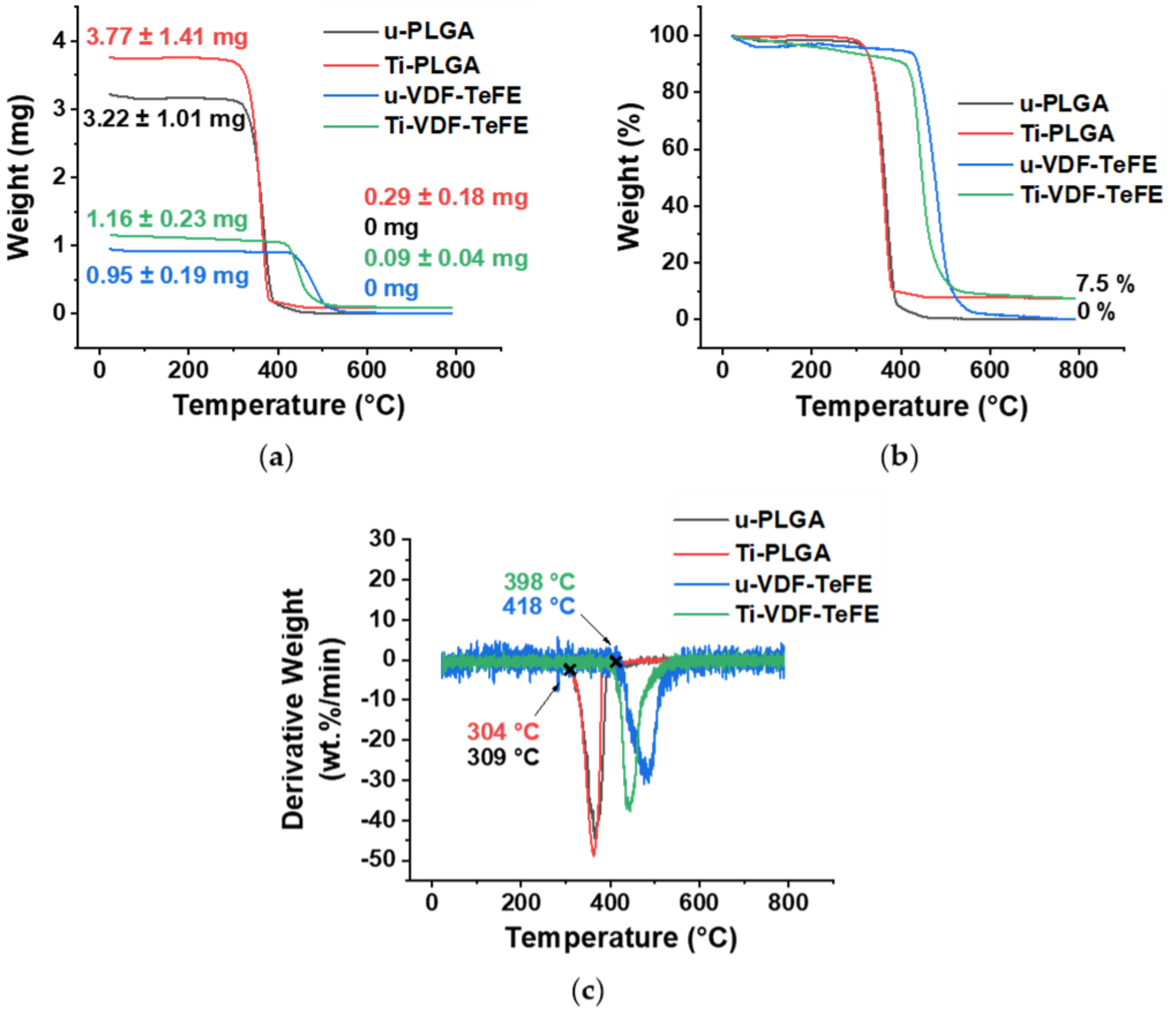

Figure S7. Thermal gravimetric analysis (TGA) and differential thermogravimetric (DTG) curves of the unmodified (u-PLGA and u-VDF-TeFE) and the surface-modified (Ti-PLGA and Ti-VDF-TeFE) scaffolds made from poly(lactide-co-glycolide) (PLGA) and vinylidene fluoride – tetrafluoroethylene copolymer (VDF-TeFE): (**a**) TGA curves with weight in milligrams (mg) vs. temperature, (**b**) TG curves with weight in percent (%) vs. temperature, (**c**) DTG curves.
